# Hide and seek: a comparative autoradiographic in vitro investigation of the adenosine A3 receptor

**DOI:** 10.1007/s00259-014-2985-2

**Published:** 2015-03-05

**Authors:** D. Haeusler, L. Grassinger, F. Fuchshuber, W. J. Hörleinsberger, R. Höftberger, I. Leisser, F. Girschele, K. Shanab, H. Spreitzer, W. Gerdenitsch, M. Hacker, W. Wadsak, Markus Mitterhauser

**Affiliations:** 1 Department of Biomedical Imaging and Image-guided Therapy, Medical University of Vienna, Waehringer Guertel 18-20/3L, 1090 Vienna, Austria; 2 Department of Biomedical Analytics, University of Applied Sciences Wiener Neustadt, Wiener Neustadt, Austria; 3 Cognitive Science Research Platform, University of Vienna, Vienna, Austria; 4 Institute of Neurology, Medical University of Vienna, Vienna, Austria; 5 Department of Drug and Natural Product Synthesis, University of Vienna, Vienna, Austria; 6 Institute of Biomedicinal Research, Medical University of Vienna, Vienna, Austria

**Keywords:** Adenosine A3 receptor, CNS, Periphery, Autoradiography, MRS1523, FE@SUPPY

## Abstract

**Purpose:**

Since the adenosine A3 receptor (A3R) is considered to be of high clinical importance in the diagnosis and treatment of ischaemic conditions (heart and brain), glaucoma, asthma, arthritis, cancer and inflammation, a suitable and selective A3R PET tracer such as [^18^F]FE@SUPPY would be of high clinical value for clinicians as well as patients. A3R was discovered in the late 1990s, but there is still little known regarding its distribution in the CNS and periphery. Hence, in autoradiographic experiments the distribution of A3R in human brain and rat tissues was investigated and the specific binding of the A3R antagonist FE@SUPPY and MRS1523 compared. Immunohistochemical staining (IHC) experiments were also performed to validate the autoradiographic findings.

**Methods:**

For autoradiographic competition experiments human post-mortem brain and rat tissues were incubated with [^125^I]AB-MECA and highly selective compounds to block the other adenosine receptor subtypes. Additionally, IHC was performed with an A3 antibody.

**Results:**

Specific A3R binding of MRS1523 and FE@SUPPY was found in all rat peripheral tissues examined with the highest amounts in the spleen (44.0 % and 46.4 %), lung (44.5 % and 45.0 %), heart (39.9 % and 42.9 %) and testes (27.4 % and 29.5 %, respectively). Low amounts of A3R were found in rat brain tissues (5.9 % and 5.6 %, respectively) and human brain tissues (thalamus 8.0 % and 9.1 %, putamen 7.8 % and 8.2 %, cerebellum 6.0 % and 7.8 %, hippocampus 5.7 % and 5.6 %, caudate nucleus 4.9 % and 6.4 %, cortex 4.9 % and 6.3 %, respectively). The outcome of the A3 antibody staining experiments complemented the results of the autoradiographic experiments.

**Conclusion:**

The presence of A3R protein was verified in central and peripheral tissues by autoradiography and IHC. The specificity and selectivity of FE@SUPPY was confirmed by direct comparison with MRS1523, providing further evidence that [^18^F]FE@SUPPY may be a suitable A3 PET tracer for use in humans.

## Introduction

Adenosine exerts its various effects via four different G-protein-coupled receptors: adenosine A1, adenosine A2A, adenosine A2B and adenosine A3 receptor (A1R, A2AR, A2BR and A3R, respectively). The most recently discovered receptor in the adenosine receptor family is the A3 subtype. Since its discovery in the late 1990s, few data regarding the distribution and density of A3R in vivo have been reported and are a matter of controversy [1]. Benarroch has stated that the A3R is present in the hippocampus and cerebellum in medium or low abundance [2]. Others have found A3R in the thalamus and hypothalamus [3], an in the rat in the hippocampus [4, 5] and cortex [6]. In the periphery, high densities of A3 mRNA have bee found in the spleen, lung, uterus and testes. Medium density has been reported in the liver and bladder, and low densities have been found in the heart, aorta, stomach, jejunum, proximal colon, kidney and eyes [7]. Furthermore, the presence of A3R has been confirmed in smooth muscle tissue of blood vessels and the aorta of rats [8].

 Since, A3R is considered to be of high clinical importance in the diagnosis and treatment of ischaemic conditions (heart and brain), glaucoma, asthma, arthritis, cancer and inflammation [9], all authors agree that there is an urgent need for a ligand with high affinity and selectivity for the visualization of A3R in vivo. Since PET allows noninvasive visualization and quantification of receptor systems in vivo, a suitable A3R PET tracer would be of high value for the evaluation of the presence of A3R, both in vitro and in vivo. Hence, we developed and introduced [^18^F]FE@SUPPY as the first PET tracer targeting A3R [10, 11]. In the meantime, other potential PET tracers including a ^76^Br-labelled compound [12], [^18^F]FE@SUPPY:2 [13, 14] and recently, ^11^C-labelled 1,2,4-triazolo[4,3-α]quinoxalin-1-one derivatives [15] have been synthesized and their affinity for A3R evaluated. So far, in successful preclinical evaluations, [^18^F]FE@SUPPY [14, 16, 17] has shown an elevated brain to blood ratio in rats [11], and metabolic stability in vitro and ex vivo in the brain for 30 min [16], indicating that [^18^F]FE@SUPPY could potentially be useful for PET imaging studies in humans.

**Fig. 1:**
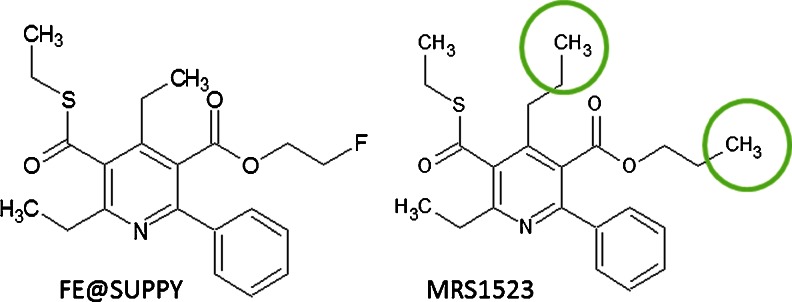
A3R antagonists FE@SUPPY and MRS1523. Chemical structures of the 1,4-dihydropyridines

Hence, as next steps in the preclinical evaluation process, in this study autoradiographic experiments were performed to determine the distribution of A3R in human brain regions (hippocampus, thalamus, cortex, caudate nucleus, putamen and cerebellum), in coronal rat brain tissues and in rat peripheral tissues (testes, heart, lung and spleen), and to compare the specific binding (SB) of the A3R antagonist FE@SUPPY (that is, the unlabelled standard compound of the A3R PET tracer [^18^F]FE@SUPPY) and the structurally related A3R antagonist MRS1523 (Fig. 1). Immunohistochemical staining (IHC) experiments were also performed to validate the autoradiographic findings.

## Materials and methods

### Materials

Human post-mortem brain tissues were collected following local regulations for diagnostic purposes. Anonymized tissue samples remaining after the diagnostic evaluation were used in this project in the context of a study (“Molecular neuropathologic investigation of neurodegenerative diseases”; collection of biological samples named “CJD KIN-Biobank”) approved by the Ethics Committee of the Medical University of Vienna (No. 396/2011) and followed the principles of the Declaration of Helsinki.

#### Autoradiographic experiments

Tissue from six different anatomical human brain regions (hippocampus, thalamus, cortex, caudate nucleus, putamen and cerebellum, *n* = 2) was quick-frozen in isopentane (2-methylbutane, CAS 78-78-4; Sigma Aldrich, Austria) at −45 °C. Four male wild-type rats (200–300 g) were deeply anaesthetized with diethylether and rapidly decapitated. Rat organs (brain, testes, heart, lung and spleen) were removed and subsequently quick-frozen as described for human tissues. Frozen tissues were cut in a microcryotome (Thermo Scientific Microm HM 560; HistoCom, Austria) into 10-μm thick tissue slices, thaw-mounted on microscope slides (Thermo Scientific, Menzel-Gläser SuperFrost® Plus; Braunschweig, Germany), dried and stored at −40 °C until processing. A barrier pen was purchased from Invitrogen (Mini PAP PEN; Zymed Laboratories, Carlsbad, CA).

 The standard compound FE@SUPPY (5-ethyl 2,4-diethyl-3-((2-fluoroethyl)sulphanylcarbonyl)-6-phenylpyridine-5-carboxylate) was synthesized at the Department of Drug and Natural Product Synthesis of the University of Vienna, Austria [19]. The A3R radioligand [^125^I]AB-MECA (NEX312; A3R agonist, 0.59 nM [20]) and the Cyclone phosphor imager (Cyclone Plus storage phosphor system) and phosphor imager plates (MS multisensitive phosphor screens long type) were purchased from Perkin Elmer (Waltham, MA), and lead-shielded autoradiography cassettes from Fisher Scientific (Pittsburgh, PA).

EDTA (ethylenediaminetetraacetic acid), NaCl, adenosine deaminase (ADA) and instant thin layer chromatography (ITLC) paper (TLC silica gel 60 RP-18) were purchased from Merck, Austria. DPCPX (CAS 102146-07-6), SCH442416 (CAS 316173-57-6), MRS1754 (MDL number MFCD11046004) and MRS1523 (MDL number MFCD03787977) and all other chemicals were purchased from Sigma Aldrich, Austria.

#### Immunohistochemical staining

Primary antibody (sc-13938, anti-adenosine A3-R) was purchased from Santa Cruz Biotechnology, CA. Secondary antibody (biotinylated anti-rabbit IgG), avidin-biotin complex (Vectastain ABC kit, PK 4001) and aqueous mounting medium (VectaMount) were obtained from Vector Laboratories, CA. DAB substrate kit (ab 94665) was purchased from Abcam (UK). Phosphate-buffered saline (PBS, pH 7.4, 10x solution) was purchased from Morphisto Evolutionsforschung und Anwendung GmbH (Germany). Hydrogen peroxide (H_2_O_2_), haemalum, ethanol and n-butyl acetate were purchased from Merck (Germany). Histofluid was obtained from Paul Marienfeld GmbH & Co. KG (Germany), cover plates (24 × 60 mm) were purchased from Menzel-Gläser HistoCom (Austria). All other chemicals were obtained from Sigma Aldrich (Austria). Optical and metric analysis of the stained tissues was performed with a Pannoramic DESK scanner from 3D-HISTECH (Hungary).

### Methods

#### Autoradiographic experiments

Tissues were surrounded with a hydrophobic barrier using a barrier pen and were preincubated at room temperature for 30 min with a 50 mM Tris-HCl buffer solution (pH 7.4) containing 1 mM EDTA, 100 mM NaCl and 2 U/ml ADA. After preincubation, the tissue sections were washed in binding buffer (50 mM Tris-HCl buffer, pH 7.4) and air-dried. Slices were then incubated directly with a solution containing the A3R radioligand [^125^I]AB-MECA (0.7 nM) and the dedicated highly selective adenosine receptor subtype blocking agents (Table 1).

**Table 1:** Selective compounds for the adenosine receptors (A_1_, A_2A_, A_2B_ and A_3_)

Compound	Receptor	Ligand	Species	Affinity (nM)	Reference	Selectivity vs. other ARs
DPCPX	A1	Antagonist	Human	nd		nd
			Rat	*K*i 0.46	[21]	740-fold vs. A2
SCH442416	A2A	Antagonist	Human	*K*i 0.48	[22]	23,000-fold vs. A1
			Rat	*K*i 0.5		nd
MRS1754	A2B	Antagonist	Human	*K*i 1.97	[23]	200-fold vs. A1, 290-fold vs. A2A, 290-fold vs. A3
			Rat	nd		nd
MRS1523	A3	Antagonist	Human	*K*i 18.9	[18]	530-fold vs. A1, 190-fold vs. A2A
			Rat	*K*i 113		140-fold vs. A1, 20-fold vs. A2A
FE@SUPPY	A3	Antagonist	Human	*K*i 4.2	[18]	nd
			Rat	*K*i 600		19-fold vs. A1, 12-fold vs. A2A
[^125^]AB-MECA	A3, A1	Agonist	Human	*K*d 0.59	[20, 24]	nd

*nd* no data

It is noteworthy that the radioligand [^125^I]AB-MECA shows affinity towards both adenosine receptors A1R and the A3R [20, 24]. The explanation for this is a 49 % sequence homology of the two proteins [25]. Therefore, to ensure complete blocking of A1R, as well as of A2AR and A2BR, the various competitors were used at concentrations 1,000-fold of their inhibition constants (*K*i) as listed in Table 1. Furthermore, the concentrations of the competitors used were also adapted to varying *K*i values according to the species differences of the A3R between rats and humans (Table 1).

In detail, the different incubation solutions for the human tissues included the radioligand [^125^I]AB-MECA (0.7 nM), DPCPX (A1R, 460 nM), SCH442416 (A2AR, 48 nM), MRS1754 (A2BR, 1.97 μM), and MRS1523 (A3R, 19 μM) or FE@SUPPY (A3R, 4.2 μM) in the binding buffer. For the rat tissues, the incubation solutions included the radioligand [^125^I]AB-MECA (0.7 nM), DPCPX (460 nM), SCH442416 (500 nM), MRS1754 (1.97 μM), and MRS1523 (113 μM) or FE@SUPPY (600 μM) in the binding buffer. Target tissues were incubated under optimum reaction conditions (60 min, room temperature) in a lead-shielded cell. After incubation, tissues were washed with buffer, rinsed with ice-cold water, and dried. Samples were then placed on the phosphor imager plates for exposure (9–14 days) in dedicated lead-shielded cassettes. Autoradiographic images were analysed with a Cyclone phosphor imager and data were analysed with OptiQuant data processing software version 5.0 and Microsoft Excel 2007.

#### Immunohistochemical staining

IHC experiments were performed according to a standard protocol [26] on vicinal tissue samples of the human and rat tissues used for autoradiography. First, the tissues were fixed for 2 min in acetone/methanol (1:1). The tissues were then incubated with 0.3 % hydrogen peroxide for 30 min to block endogenous peroxidase activity and incubated with an appropriate blocking serum (goat) for 30 min to prevent nonspecific binding (NB). The tissue slices were then incubated with the primary antibody (rabbit) diluted 1:100 for 17 h at 4 °C. The tissue slices were incubated for 30 min with biotinylated secondary antibody (anti-rabbit) and the avidin biotin complex prepared according to the manufacturer’s instructions. The slices were then incubated for 1–2 min with freshly prepared DAB solution. Tissues were washed twice with PBS for 5 min between each step, except after the incubation with the blocking serum. As a reference in each tissue, the nuclei were additionally stained with haemalum. To achieve permanent staining, tissues were dehydrated with alcohol and n-butyl acetate and covered with Histofluid. As a positive control, rat testicular tissue was stained in every IHC experiment. As a negative control each tissue was stained in parallel with and without antibody (with binding buffer to compensate for the amount of liquid). Finally, the tissues were scanned and analysed using a Pannoramic DESK scanner.

### Statistical analysis

#### General

All values are given as arithmetic means ± standard deviation. To determine the significance of differences, a two-tailed *t* test with *α* = 0.95 was performed using the statistics add-on in Windows Excel 2010. Values of *P* ≥ 0.05 were considered not significant.

#### Analysis of the phosphor imager values

The phosphor imager software (OptiQuant) expresses the radioactive signal from the probes in digital light units per square millimetre (DLU/mm^2^). The intensity of the light from the stored energy is proportional to the amount of activity in the sample. Therefore, aliquots of five concentrations of radioligand stock solutions were spotted onto ITLC paper, allowed to dry and placed on the phosphor film. After scanning, a calibration curve based on the concentrations of the aliquots was established. Then, the amount of standards (kilobecquerels) and the phosphor imager signals (DLU/mm^2^) derived from the probes were correlated. With the known specific activity of the radioligand and the equation from the linear regression curve (*y* = *kx* + *d*), the corresponding relative concentration (femtomoles per square millimetre) of receptor was calculated. To receive the DLU/mm^2^ values from the probes, regions of interest (ROIs) were drawn for each tissue of each experiment. DLU/mm^2^ values were normalized to percentage values to enable comparison of the data from the different experiments.

**Fig. 2:**
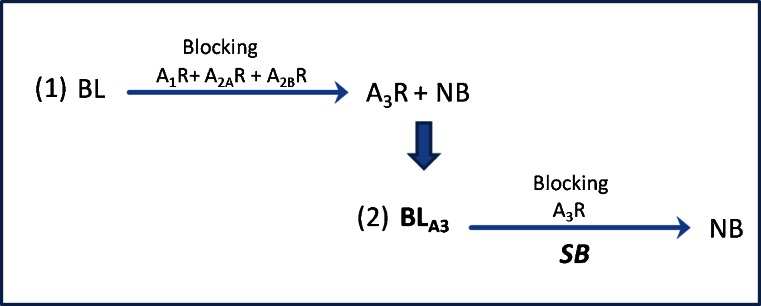
“Two-step” analysis scheme of the comparative autoradiographic experiments (*BL* baseline binding, *NB* nonspecific binding, *BL*_*A3*_ remaining A3R and nonspecific binding, called “Baseline A3”)

Consequently, autoradiographic experiments were analysed in a “two-step” scheme as shown in Fig. 2. In detail, in step 1 baseline values (reflecting the total binding of the radioligand on the tissue) were set to 100 %, and competition was expressed relative to this value. After blocking of the A1/A2 receptors (A1R, A2AR, A2BR) the A3R binding and NB was left. Therefore, in step 2, further blocking with the A3R-selective antagonists MRS1523 or FE@SUPPY led to further competition of the radioligand leaving NB remaining.

The following equations were used for analysis of the data:

$$ \begin{array}{l}\mathrm{B}\mathrm{L}=100\%=\mathrm{total}\ \mathrm{binding}\ \mathrm{of}\ \mathrm{the}\ \mathrm{radioligand}\ \left[{}^{125}\mathrm{I}\right]\mathrm{AB}\hbox{-} \mathrm{MECA} = \mathrm{A}1+\mathrm{A}2\mathrm{A}+\mathrm{A}2\mathrm{B}+\mathrm{A}3+\mathrm{N}\mathrm{B}\hfill \\ {}\hfill \mathrm{B}\mathrm{L}-\left(\mathrm{A}1 + \mathrm{A}2\mathrm{A} + \mathrm{A}2\mathrm{B}\right) = \mathrm{A}3 + \mathrm{N}\mathrm{B} = {\mathrm{BL}}_{\mathrm{A}3} = \mathrm{B}\mathrm{aseline}\ \mathrm{A}3\hfill \\ {}\hfill {\mathrm{BL}}_{\mathrm{A}3} - \mathrm{A}3 = \mathrm{N}\mathrm{B}\hfill \end{array} $$

Thus, SB of the two A3R antagonists was characterized indirectly through competition, and was calculated as follows:

$$ \begin{array}{l}\hfill \mathrm{S}\mathrm{B}=\mathrm{B}\mathrm{L}-\mathrm{N}\mathrm{B}\hfill \\ {}\hfill \mathrm{S}\mathrm{B}=\left(\mathrm{A}1 + \mathrm{A}2\mathrm{A} + \mathrm{A}2\mathrm{B}\right)-\mathrm{N}\mathrm{B}=\mathrm{specific}\ \mathrm{A}3\ \mathrm{binding}\ \mathrm{in}\ \mathrm{relation}\ \mathrm{t}\mathrm{o}\ \mathrm{B}\mathrm{aseline}\hfill \end{array} $$

#### Analysis of the IHC tissues

A3R antibody-stained tissues were analysed visually using a Panoramic DESK scanner with a 40-fold magnification. Each tissue was compared with a corresponding negative control tissue without antibody. For differentiation of nuclei and other cell compartments, additional haemalum staining was conducted. Rat brain tissues were correlated with a rat brain atlas (Paxinos) for determination of bregma and identification of the different brain areas.

## Results

### Autoradiographic experiments

An general overview of the A3R (expressed as SB and relative receptor density) obtained with the A3R antagonists MRS1523 and FE@SUPPY in human brain, rat brain and rat peripheral tissues is given in Table 2. Statistical analysis (*t* tests) revealed no significant differences between the two compounds.

**Table 2:** Overview of SB of the A3R obtained with the antagonists MRS1523 and FE@SUPPY vs. [^125^I]AB-MECA on rat and human tissue origin

Species	Tissue	Specific binding (%)		Relative receptor density (fmol/mm^2^)	ARs^a^
		MRS 1523	FE@SUPPY		
Human	Hippocampus	5.7 ± 0.3	5.6 ± 0.7	0.39	A1, A3
	Thalamus	8.9 ± 4	9.1 ± 1	0.79	A1, A2A, A3
	Cortex	4.9 ± 1	6.3 ± 4	0.40	A1, A2A
	Caudate nucleus	4.9 ± 2	6.4 ± 3	0.43	A1, A2A
	Putamen	7.8 ± 0.3	8.2 ± 1	0.84	A2A, A1
	Cerebellum	6.0 ± 2	7.8 ± 1.2	0.50	A2A, A1
Rat	Brain	5.9 ± 1.5	5.6 ± 1.2	0.39	A1–3
	Testes	27.4 ± 6	29.5 ± 3	1.20	A3 medium to high
	Heart	39.9 ± 6	42.9 ± 4	3.10	A3 medium to low
	Spleen	44 ± 10	46.4 ± 11	6.00	A3 medium to high
	Lung	42.5 ± 4	45.0 ± 5	2.60	A3 medium to high

Values are means ± standard deviations from quadruple analyses from two to four different experiments each (*n* ≤ 16)

^a^From Ribeiro et al. [45]

#### Human brain regions

**Fig. 3:**
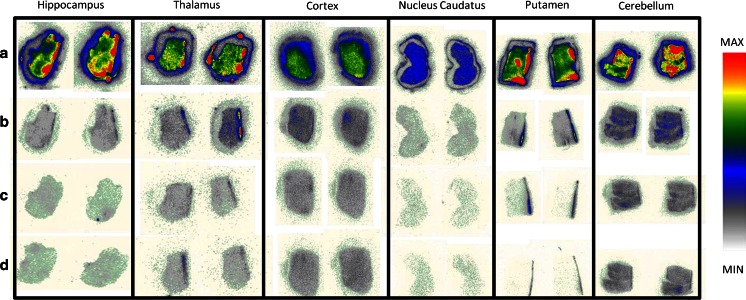
Representative autoradiographic images of [^125^I]AB-MECA (0.7 nM) binding under baseline and blocking conditions in six different human brain regions (*ed* highest uptake, *white/grey* complete blocking): **a** baseline; **b** baseline A3 (i.e. after blocking of A1, A2A, A2B; see Fig. 2); **c** additional blocking of the A3R with 19 μM MRS1523; **d** additional blocking of the A3R with 4.2 μM FE@SUPPY. Note that the “activity circle” surrounding the brain tissues is caused by tracer uptake by the lipophilic barrier pen; therefore the ROIs were drawn inside precisely around the tissues of interest

Autoradiographic images of [^125^I]AB-MECA competition under baseline and blocking conditions of six different human brain regions (hippocampus, thalamus, cortex, caudate nucleus, putamen and cerebellum) are shown in Fig. 3. High densities of A1R and A2Rs were shown in all regions by drastic decreases in radioligand binding after blocking with these receptors (Fig. 3b). Further blocking of the A3R led to the SB values given in Table 2.

#### Rat tissues

**Fig. 4:**
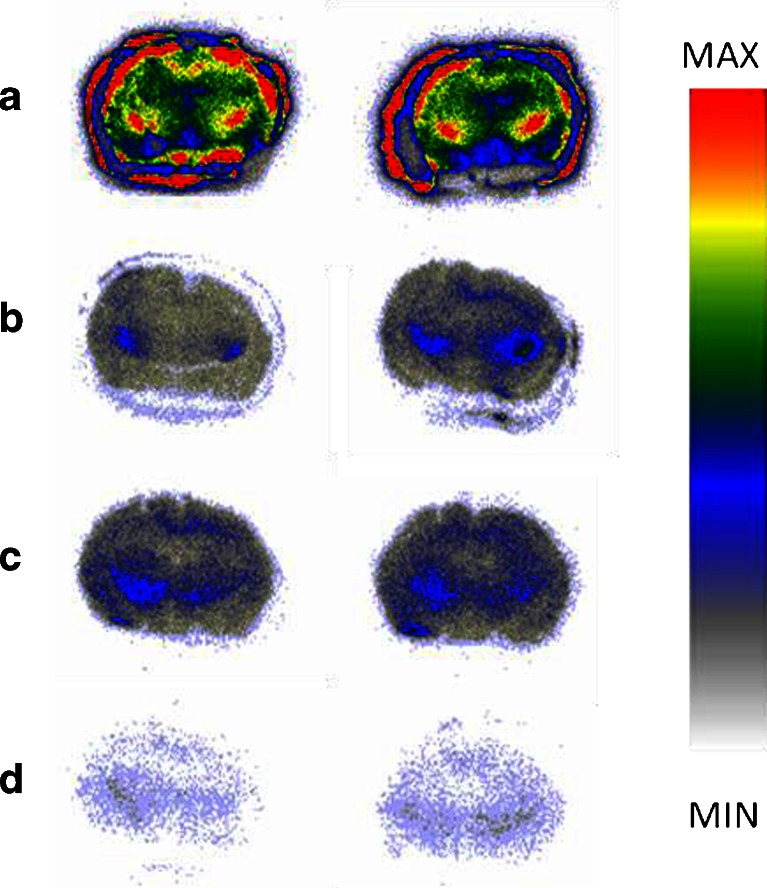
Representative autoradiographic images of [^125^I]AB-MECA (0.7 nM) binding in coronal rat brain (bregma −2,30) under baseline and blocking conditions. High blockable uptake was found in the hippocampus, striatum/caudate putamen, medial globus pallidus (*red* highest uptake, *white/grey* full competition): **a** baseline; **b** baseline A3 (i.e. after blocking of A1, A2A, A2B; see Fig. 2); **c** additional blocking of the A3R with 113 μM MRS1523; **d** additional blocking of the A3R with 600 μM FE@SUPPY. Note that the “activity circle” surrounding the brain tissues is caused by tracer uptake by the lipophilic barrier pen; therefore ROIs were drawn inside precisely around the tissues of interest

Autoradiographic images of [^125^I]AB-MECA competition under baseline and blocking conditions on rat brain tissues are shown in Fig. 4.

**Fig. 5:**
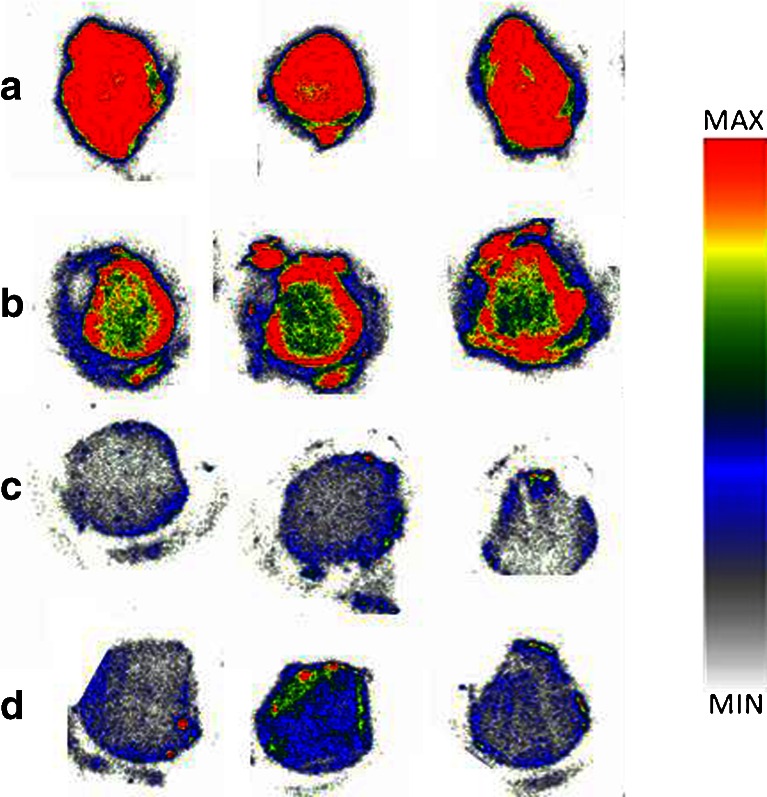
Representative autoradiographic images of [^125^I]AB-MECA (0.7 nM) binding under baseline and blocking conditions in rat testes (*red* highest uptake, *white/grey* full competition): **a** baseline; **b** baseline A3 (i.e. after blocking of A1, A2A, A2B; see Fig. 2); **c** additional blocking of the A3R with 113 μM MRS1523; **d** additional blocking of the A3R with 600 μM FE@SUPPY. Note that the “activity circle” surrounding the brain tissues is caused by tracer uptake by lipophilic barrier pen; therefore ROIs were drawn inside precisely around the tissues of interest

Autoradiographic images of [^125^I]AB-MECA competition under baseline and blocking conditions on rat peripheral tissues (testes) are shown in Fig. 5.

### Immunohistochemical staining

**Fig. 6:**
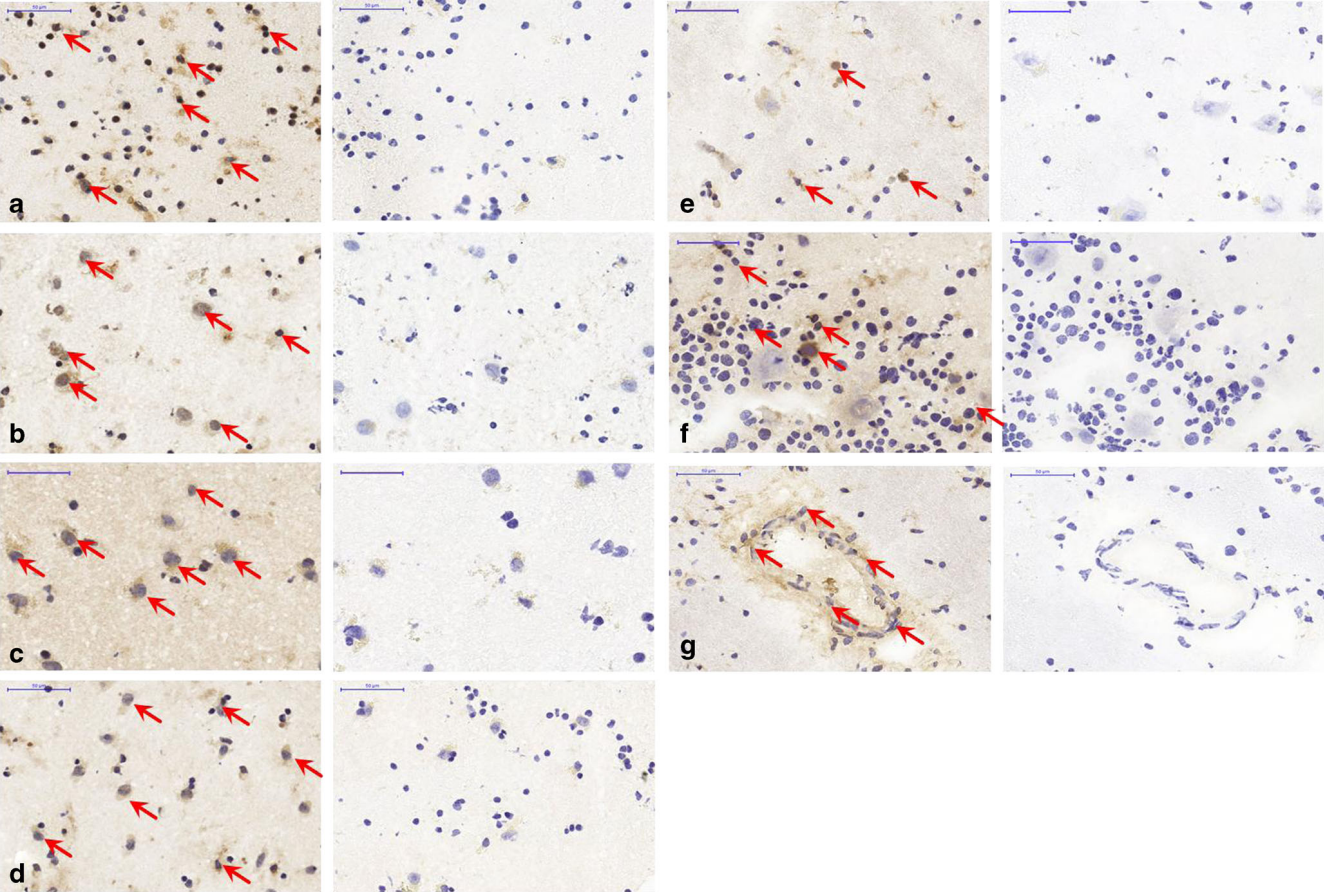
Immunohistochemical staining of A3R in human brain regions (*n* = 3–5): **a** hippocampus, **b** cortex, **c** caudate nucleus, **d** putamen, **e** thalamus, **f** cerebellum, **g** intracerebral artery, thalamus. *Left of each image pair* A3R antibody staining indicated by *red arrows*; *right of each image pair* control without A3R antibody; *blue* nuclei resulting from haemalum staining (×40, *scale bars* 50 μm)

**Fig. 7:**
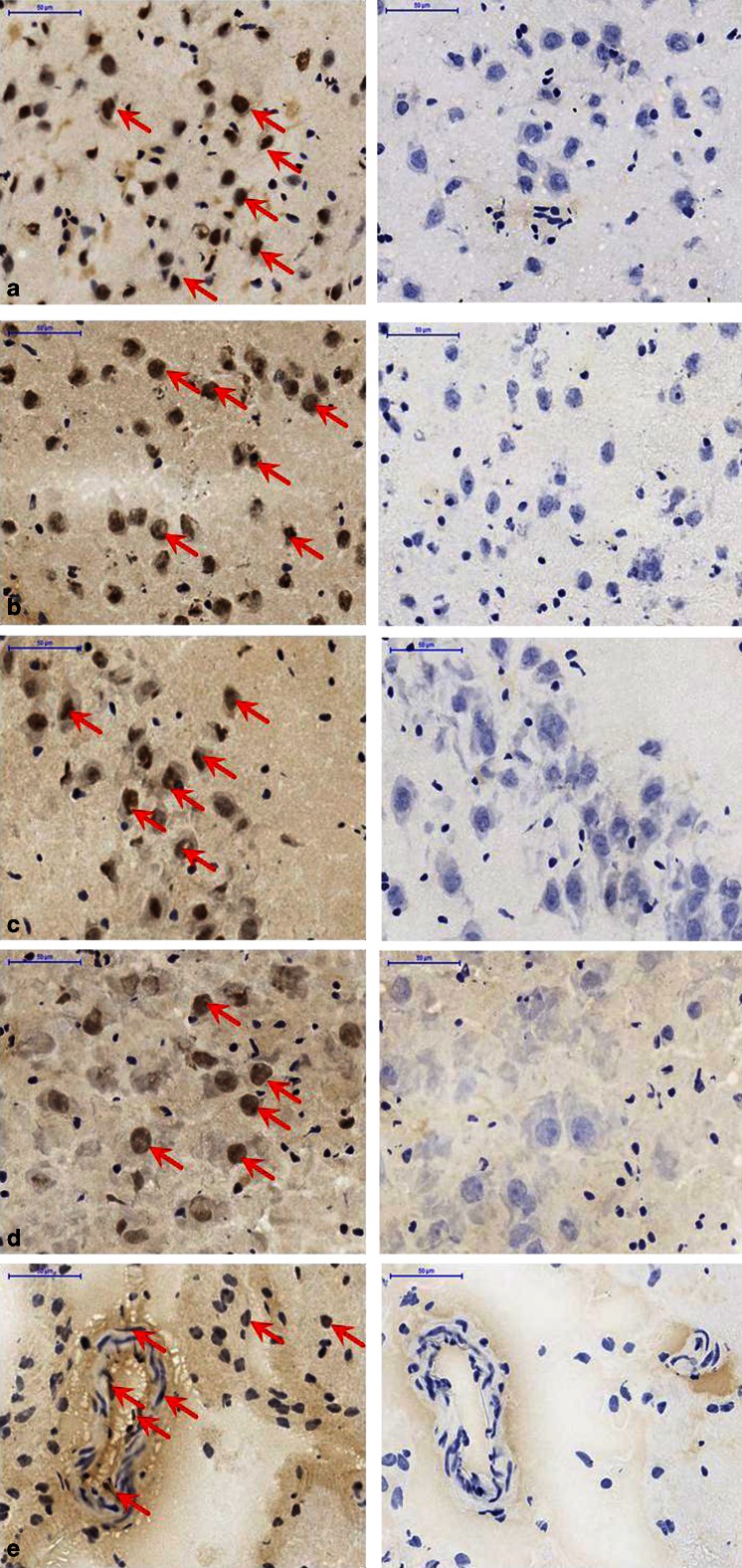
Immunohistochemical staining of A3R in rat brain regions (*n* = 3–5): **a** bregma 2,70 (nucleus accumbens), **b** bregma 4,70 (lateral olfactory tract), **c** bregma −6,30 (hippocampal fissure), **d** bregma −10,04 (superior cerebellar peduncle), **e** bregma −6,30 (artery from the hilus of dentate gyrus). *Left of each image pair* A3R antibody staining indicated by *red arrows*; *right of each image pair* control without A3R antibody; *blue* nuclei resulting from haemalum staining (×40, *scale bars* 50 μm)

**Fig. 8:**
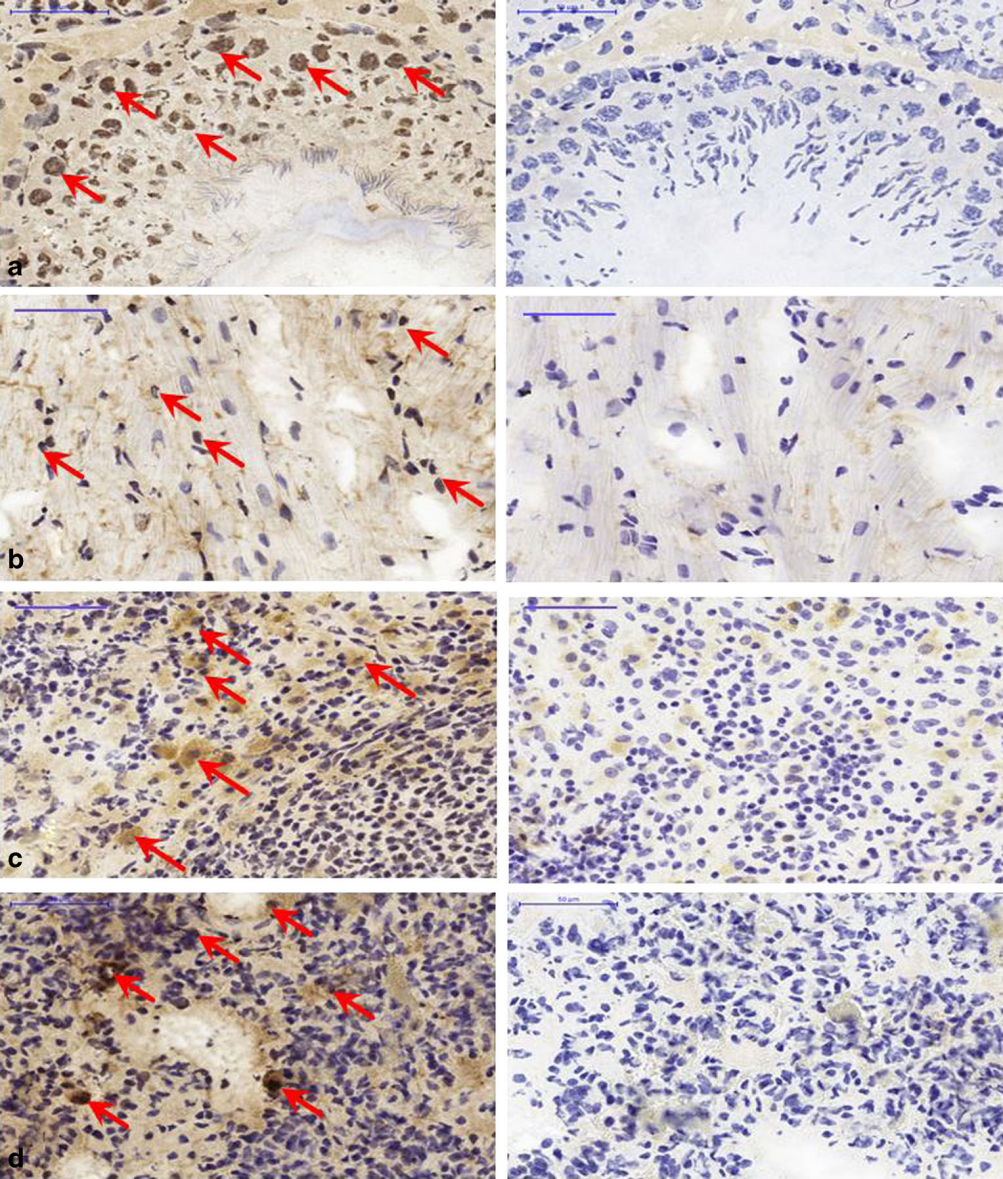
Immunohistochemical staining of A3R in rat peripheral tissues (*n* = 3–5): **a** testes, **b** heart, **c** spleen, **d** lung. *Left of each image pair* A3R antibody staining indicated by *red arrows*; *right of each image pair* control without A3R antibody; *blue* nuclei resulting from haemalum staining (×40, *scale bars* 50 μm)

A3R antibody staining (*n* = 3–5) 3–5 different staining experiments in human brain (Fig. 6), rat brain (Fig. 7) and rat peripheral tissues (Fig. 8) showed staining in all tissues. As a reference within each tissue, the nuclei were additionally stained with haemalum.

## Discussion

### General

A3R is highly overexpressed in cancer cell lines and cancer tissues [27–32] and in human enteric neurons [33] but not in the majority of normal tissues [34]. Hence, its role as a tumour marker and a target for tumour intervention and imaging is undisputed [34, 35]. Furthermore, A3 antagonists are currently discussed as a potential new approach to the treatment of cancer [9, 31, 32]. The A3Rs are also known to be involved in cardiac diseases [36], cerebral ischaemia [37], glaucoma [38], stroke [39] and epilepsy [40]. Hence, the value of A3 antagonists as pharmaceutical tools is obvious and a dedicated PET tracer for in vivo visualization A3Rs would be of high value for clinicians as well as patients.

Generally, it is well known that A3R is expressed at low levels in the whole organism [41], as well as in the brain [1–6], especially compared to the high expression rates of the A1R and A2AR in the CNS. According to the literature, A1R is abundant in most regions of the brain, with high densities in the hippocampal area, cerebellum and cortex, and A2AR has high densities in the basal ganglia, nucleus accumbens and olfactory tubercle [42]. Furthermore, A3R differs widely between humans and rats, with only 72 % homology of the two proteins [43], in contrast to general rat/human protein homologies of about 90–98 %. It is well known that 1,4-dihydropyridines (such as MRS1523 and FE@SUPPY) display rather low affinities towards the rat A3R in comparison to the human A3R. However, according to Müller and Jacobson, MRS1523 can be considered a “useful tool” amongst the A3R antagonists [44]. FE@SUPPY is structurally closely related to MRS1523 (Fig. 1) with a lower Ki towards the rat A3R and a higher Ki for the human A3R (Table 3).

**Table 3:** Selectivity and specificity data of both compounds [18]

Receptor Selectivity	*MRS1523 Ki[nM]*	*FE @ SUPPY Ki [nM]*
A3R_h_	18.9	4.2
A3R_r_	113	600
A1R_h_	10,000	n.d.
A1R_r_	15,600	11,500
A2AR_h_	3,660	n.d.
A2AR_r_	2,050	7,300

*n.d.* no data, *r* rat receptor, *h* human receptor

In summary, the low expression rate of A3R in vivo, the differences in A3R protein between humans and rats, and its reported “enigmatic” behaviour, as well as the results of some other studies and the conclusions drawn by their authors as to the whereabouts of A3R, create the impression that A3R seems to be playing hide and seek. Hence, in the present autoradiographic study we investigated the distribution and presence of the A3R protein in the CNS and periphery using two A3R antagonists, MRS1523 and FE@SUPPY, and validated the findings by IHC with a dedicated A3 antibody as a specific imaging method.

### Autoradiographic experiments

The autoradiographic experiments showed the presence of A3R in all tissues examined. Using the scheme shown in Fig. 2, A3R SB values for MRS1523/FE@SUPPY in human brain regions were calculated (Table 2). The *P* values for SB in human brain ranged from 0.4 to 0.8, in rat brain the *P* value was 0.9, and in rat peripheral tissues the *P* values ranged from 0.5 to 0.8. Since all the tissues showed significant differences between MRS1523 and FE@SUPPY throughout the whole autoradiographic set-up, we conclude that the two ligands are comparable in regard to their binding and blocking behaviour.

#### Human brain regions

**Fig. 9:**
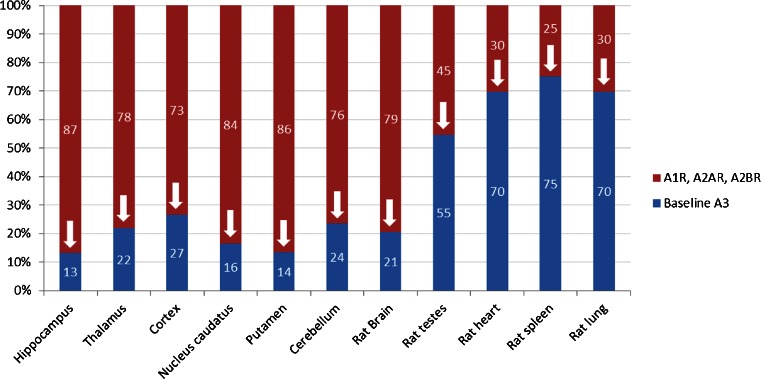
Reductions in binding of [^125^I]AB-MECA from 100 % (total binding) to “Baseline A3”. The *red* parts of the bars indicate the amounts of A1R and A2R; the corresponding *blue* parts indicate the remaining amounts of radioligand (i.e. remaining A3R and nonspecific binding, defined as “Baseline A3”). The data presented are the means of three or four autoradiographic experiments

In human post-mortem brain, blocking the A1, A2A and the A2B receptors drastically reduced the binding of [^125^I]AB-MECA to 13–27 %, respectively (Fig. 9). This decrease in radioligand binding can be explained by the low A3R/A1R selectivity of [^125^I]AB-MECA and the high abundance of the A1R in most brain regions. Blocking A3R with the selective A3R antagonist MRS1523 or with FE@SUPPY led to a further specific competition of the radioligand in all human brain regions examined. Since all other ARs which could have interfered were already blocked, this further reduction of radioligand binding reflects selective A3R competition of the radioligand, and therefore the presence of the A3R protein in the tissues of interest (Table 2).

In detail, the hippocampus, a region widely considered to show moderate to low densities of A3R [2], showed a moderate density of A3R in this study. The thalamus is considered by some to show a high density of A3R [3], which is in accordance with this study in which the thalamus showed the highest density of A3R. The observed medium to low density of A3R in the cortical tissues is partly in accordance with the literature, where cortex is sometimes even considered as a nontarget region for A3R. In contrast, we found A3R in the basal ganglia (caudate nucleus and putamen). In accordance with Ribeiro et al. [45], we found medium amounts of A3R in the cerebellum (Table 2). As shown in Fig. 3, white matter regions including the cortex and cerebellum seemed to accumulate slightly more radiotracer (leading to more baseline and background activity in the tissues). Both regions showed the highest amounts of Baseline A3 (27 % and 24 %, respectively) as shown in Fig. 8, indicating lower A1 densities than in the grey matter brain regions. Nevertheless, since A3R (and A1R) are present in both, conclusive delineation of the abundance of A3R between white and grey matter is not possible at this point. However, the A3R antagonists revealed the presence of A3R in the white matter, and this may indicate the potential for imaging neurological diseases involving the white matter, as suggested by González-Fernández et al. who found that activation of A3Rs may induce white matter death [46].

#### Rat tissues

In rat brain, blocking of A1, A2A and A2B receptors led to a drastic reduction (as observed in human brain tissues) in the total binding of [^125^I]AB-MECA to 21 % remaining activity defined as “Baseline A3” (Fig. 9). Interestingly, in the rat peripheral organs the reduction in radioligand binding was less pronounced, ranging from 25 % to 45 % (Fig. 9), the reason for which is as explained in the previous section. MRS1523 and FE@SUPPY led to an additional decrease in binding from Baseline A3 leading to the SB values given in Table 2. In the periphery, the highest presence of A3 was found in the spleen, followed by the lung, heart and testes. A3 was also found in all these tissues by Dixon et al. at the mRNA level [7].

### Immunohistochemical staining

Optical analysis of the A3 antibody-stained tissues revealed A3 in all central and peripheral regions examined. These findings are in accordance and direct correlation with the autoradiographic experiments and with literature [7]. In detail, both protein level imaging methods (IHC and autoradiography) confirmed the presence of A3 in the following regions in both humans and rats: hippocampal area, cerebellar region, and basal ganglia including the caudate nucleus and putamen. In rat brain, A3R antibody staining was found in the hippocampus, cerebellum and cingulate cortex (Fig. 6), as well as staining of intracerebral arteries (Fig. 7), which is in accordance with the findings of Di Tullio et al. who specifically identified A3R in rat pial and intracerebral arteries [47]. Interestingly, in human brain regions, intracerebral arteries also showed specific A3 staining, as seen for example in the thalamic region (Fig. 6). These findings are in accordance with the well-known fact that the A3R is involved in the central regulation of arterial blood pressure [8, 48].

## Conclusion

In summary, if one looks thoroughly into the literature, A3R sometimes appears to play hide and seek. Different findings in regard to the presence and density of the A3R have been shown by – amongst others – various in vitro methods, where different targets are measured (e.g. A3 mRNA vs. A3R protein). As is well known, data from mRNA do not necessarily reflect the presence of expressed protein. In this study, using in vitro autoradiography and IHC, the A3R protein itself was visualized and quantified.

The comparative autoradiographic evaluation of the A3R in human post-mortem tissues from six different brain regions, and rat coronal brain and peripheral tissues revealed the reliably measurable presence of A3R in all investigated tissues. The specific A3R binding of the two A3R antagonists MRS1523 and FE@SUPPY in CNS and peripheral tissues showed high correlations in all regions (Table 2). Hence, we conclude that the two ligands can be considered comparable in regard to their selectivity and specificity for A3R binding.

Since A3R overexpression is associated with a variety of diseases including cancer and neurological pathologies, there is an urgent need for a ligand with high affinity and selectivity for this receptor. The findings of this and previous studies indicate the potential of FE@SUPPY in this role, and provide further evidence to support the use of [^18^F]FE@SUPPY as a specific PET tracer for the noninvasive visualization of A3R-related pathologies. However, we are of the view that in vivo studies of [^18^F]FE@SUPPY in animal models have to be performed before its use in humans (e.g. for tumour imaging) can seriously be recommended.
